# Stool-Based Tests for Colorectal Cancer Screening: Performance Benchmarks Lead to High Expected Efficacy

**DOI:** 10.1007/s11894-020-00770-6

**Published:** 2020-06-03

**Authors:** Derek W. Ebner, John B. Kisiel

**Affiliations:** Division of Gastroenterology and Hepatology, Mayo Clinic, Rochester, MN USA

**Keywords:** Colonic neoplasms/prevention, Fecal immunochemical test, Occult blood/methods, Multi-target stool DNA test, Liquid biopsy

## Abstract

**Purpose of Review:**

Participation goals for colorectal cancer (CRC) screening in the USA have not been met. Non-invasive screening strategies may improve CRC screening participation. We highlight recent literature on stool-based screening performance and expectations for emerging non-invasive screening tests.

**Recent Findings:**

Stool-based CRC screening detects screen-relevant colorectal neoplasia and outperforms a currently available plasma assay. Though modestly sensitive for CRC, adherence to annual fecal immunochemical testing (FIT) is sub-optimal. Multi-target stool DNA (MT-sDNA) has greater adherence, superior sensitivity for screen-relevant lesions (including those in the proximal colon and sessile serrated architecture), and equivalent specificity to FIT over a 3-year period.

**Summary:**

Stool-based CRC screening tests are anticipated to reduce the incidence and mortality of CRC through detection of early-stage cancers and high-risk polyps. These endpoints in performance will need to be met by emerging blood sample–based tests in order have meaningful impact in clinical practice.

## Introduction

In the USA, the death rate from colorectal cancer (CRC) has declined by 54% since 1970; ample evidence suggests that organized screening has played a major role in this considerable achievement [1–3]. Despite this progress, over 100,000 new cases of colon cancer and roughly 53,000 related deaths will occur this year in the USA, where CRC is the second leading cause of cancer-related death in men and women combined [1]. Globally, CRC is the second leading cause of cancer-related death and the incidence of CRC is increasing, particularly in countries with rising rates of saturated fat intake and/or smoking [4]. To prevent CRC, stool-based screening with a fecal immunochemical test (FIT) is most commonly used worldwide [5]; screening colonoscopy is used mostly in Germany and the USA.

Unlike a non-invasive screen, structural examinations (colonoscopy and flexible sigmoidoscopy) have the capacity to perform polypectomy; these enable removal of precursor lesions and lower the incidence and mortality of CRC [6]. For example, in a prospective randomized control study, those offered a one-time screening by flexible sigmoidoscopy had a decreased incidence and mortality of CRC by 33% and 43%, respectively; this benefit with flexible sigmoidoscopy is mostly confined to the left colon [7, 8], where fewer than half of incident cases may occur [9]. In observational studies, colonoscopy also favorably reduces the incidence (− 56%) and mortality (− 68%) of CRC; however, this benefit was significantly greater in the left colon than the right [8].

Despite the reductions in CRC incidence and mortality from screening, adherence to CRC screening recommendations remains poor. It is estimated that nearly 1/3 of eligible adults go unscreened for CRC [10••]. While absence of insurance or a regular health care provider are major barriers [11], adherence to structural screening tests is sub-optimal among those with access to care due to required time off of work, cost of structural screening, scheduling of the exam, bowel preparation, and fear of procedural pain/risks [12, 13]. Non-invasive tests may be an attractive option for those who find these features of colonoscopy prohibitive. Controlled trial data suggest that when patients are offered a choice between structural (colonoscopy) and non-invasive screening (FIT), adherence rates are significantly higher than when referred for screening colonoscopy as the only modality [14].

When assessing the benefits of structural versus non-invasive screening for CRC, it is important to recall that detection of curable-stage cancers will potentially lower CRC mortality; only tests that can detect precursors (and result in their ultimate removal) will lower CRC incidence. For example, annual guaiac-based fecal occult blood testing (gFOBT) has been shown in randomized controlled trials to lower the mortality from CRC by 14% over a 10-year period, compared with no screening but does not reduce CRC incidence [15, 16]. In contrast, newer generation occult blood testing by FIT detects early-stage cancer and some screen-relevant pre-cancers, including advanced adenomas (adenomatous polyp ≥ 1 cm or containing > 25% villous component or high-grade dysplasia). A recent review evaluated the results of CRC screening programs in Europe; for a positive FIT, the reported positive predictive value for advanced adenomas ranged from 5% in Ireland to 30% in Italy [5]. However, serrated polyps ≥ 1 cm are poorly detected as are all other screen-relevant lesions in the proximal colon [5]. Most recently, advances in technology have brought new non-invasive options; these include the multi-target stool DNA (MT-sDNA) (Cologuard®, Exact Sciences Corporation, Madison, WI) test and the methylated *SEPTIN9* (*SEPT9*) blood plasma test (Epi proColon®, Epigenomics, San Diego, CA). MT-sDNA detects curable-stage CRC with high sensitivity 93% (95% CI, 84–98%) to 100% (69–100%) and outperforms FIT in the detection of both advanced adenomas and serrated precursors with sensitivity increasing in association with risk of progression to cancer [17–19]. *SEPT9* sensitivity for CRC appeared high in case-control studies but showed lesser performance in asymptomatic screening patients, in whom precursor sensitivity was less than historically seen with FIT [20]. With few studies directly comparing these non-invasive CRC screening options and no randomized controlled trial data on effectiveness, we sought to critically assess the available comparative literature on these tests. We further review indirect projections of test efficacy to guide clinicians caring for patients seeking an alternative to screening colonoscopy.

## Overview of Stool-Based CRC Screening Tests

While there are multiple commercially available products, stool testing broadly falls into three platforms: gFOBT, FIT, and MT-sDNA (Table 1). None of these options requires a cathartic bowel-prep or anesthesia and is conducted at home. Both gFOBT and FIT require annual evaluation; MT-sDNA is recommended for every 3-year use by the manufacturer and is also supported by an Archimedes model (Archimedes Inc., San Francisco, CA) study that used a 200,000 patient population and found a reduction in CRC incidence and mortality by 57% and 67% respectively at 3-year interval testing [21]. Additionally, a > 2400 person observational study of the positive and negative predictive values of MT-sDNA at 3-year intervals has now closed to enrollment (ClinicalTrials.gov Identifier: NCT02419716).

**Table 1:** Characteristics of commercially available non-invasive tests for CRC screening

Test	Frequency	Evidence of efficacy	Other factors
Stool-based			
gFOBT	Every 1 year	•Randomized controlled trials show reduction in mortality by 14%	•Requires dietary and medication restrictions •3 separate at-home collections
FIT	Every 1 year	•Cross-sectional studies show higher sensitivity that gFOBT •Modeling studies show comparable benefits and lower harms compared to colonoscopy •Less sensitive for lesions in the right colon^†^	•Single specimen collected at home •Adherence benefits from patient navigation (at provider cost)
MT-sDNA	Every 3 years^††^	•Direct cross-sectional comparison studies show superiority to FIT for curable-stage CRC, advanced adenoma, and advanced sessile serrated polyps •Equally sensitive for lesions in the right and left colon •Modeling studies show comparable benefits and harms to FIT	•Single specimen collected at home •Patient navigation included •Diagnostic colonoscopy is sufficient to evaluate all positive results •Specificity comparable to FIT performed for 3 years
Blood-based			
*SEPTIN9*	Not established	•Direct comparison shows significantly lower specificity than FIT for CRC •Direct comparison shows lower sensitivity for CRC and precursors vs. MT-sDNA	•Assayed from blood plasma; collection cannot be done at home •Not endorsed by guidelines* •Limited insurance coverage

^†^Defined as proximal (right) or distal (left) to the splenic flexure

^††^Manufacturer recommendation

*Guaiac-based fecal occult blood test (*gFOBT*), fecal immunochemical test (*FIT*), and multi-target stool DNA (*MT-sDNA*) are all endorsed by the United States Preventive Services Task Force (2016), Multi-Society Task Force on Colorectal Cancer Screening (2017), and American Cancer Society (2015)

Peroxidase substances in fruits and vegetables and dietary heme will cross-react with the gFOBT assay; these must not be consumed for at least 3 days prior to the start of collection of three separate individual stool samples. FIT does not require dietary modification and utilizes a single stool sample. As reviewed below, FIT is also substantially more accurate than gFOBT and has largely replaced the use of gFOBT in clinical practice. FIT has many different manufacturers, each with slightly different assay performance characteristics. This is particularly attractive in countries with limited capability to perform colonoscopy [5]. No consensus for optimal threshold of hemoglobin detection by FIT has been determined. A threshold between 20 and 30 μg/g has been proposed for most countries whose colonoscopy capability can meet the anticipated 5% positivity rate for FIT at this threshold [22]. The American Society for Gastrointestinal Endoscopy consensus statement on FIT favors a lower threshold cut-off to define a positive test, ≤ 20 μg/g, and reports an expected test performance of 80% sensitivity for CRC and 20–30% sensitivity for advanced neoplasia detection [23••]. A more recent systematic review explored the influence of different hemoglobin level thresholds to generate positive or negative calls on FIT performance [24••]. In their review, Imperiale et al. examined various thresholds spanning from ≤ 10 to ≥ 20 μg/g and found the greatest sensitivity to be at a threshold of 10 μg/g. At this level of detection, CRC sensitivity was 91% (84–95%). Advanced adenoma detection by the FIT assays overall ranged from 25% (20–31%) to 40% (33–47%). Importantly, at a positive testing threshold between 10 and 20 μg/g, a 7% false positive rate is expected [24••]. While FIT performance characteristics have usually been reported from clinical trials using colonoscopy as the criterion standard, this design is not always followed. In study settings where cost is a major constraint and FIT widely adopted in clinical practice, the positive predictive value of FIT has been measured by performing colonoscopy only on those with positive FIT; FIT-negative patients were followed with clinical registries to estimate unmeasured performance characteristics. Unfortunately, this approach has recently been shown to overestimate FIT performance due to potential bias in the modeling calculations used to report sensitivity and specificity and most notably overestimates sensitivity [25••].

Like FIT, MT-sDNA does not require modification of diet prior to use and assays one stool collection. While all three stool-based strategies measure occult blood, MT-sDNA also quantifies DNA targets exfoliated from CRC and precursors [26]. These DNA components include methylated *BMP3* and *NDRG4* and mutant *KRAS*; *ACTB* is assayed to account for human DNA content in each sample, and results are reported as positive or negative based on a composite score generated by a validated, multi-parameter algorithm [27].

## FIT and MT-sDNA: Comparative Accuracy

While the performance of FIT has been compared to colonoscopy in many studies, as reviewed above, there are only three studies that have directly compared FIT to MT-sDNA and colonoscopy (Table 2) [17–19]. These are critically important as this is the only way to assess the relative performance of these two commonly used stool tests. In the only study sufficiently powered to show a difference in CRC sensitivity (“DeeP-C” ClinicalTrials.gov number, NCT01397747), MT-sDNA was superior to FIT for detection of any CRC or curable-stage CRC with a reported sensitivity of 92% (83–98%) to 93% (84–97%). To detect one colorectal cancer, 166 people would need to undergo screening with MT-sDNA whereas 208 people would be required to undergo FIT [17••]. In all 3 studies, MT-sDNA showed significantly higher sensitivity for advanced pre-cancerous lesions. MT-sDNA is also more sensitive for advanced sessile serrated polyps [17, 19]. With colonoscopy as the criterion standard, the sensitivity for detecting advanced precursors with MT-sDNA was found to range between 41% (30–53%) and 48% (37–59%) [18, 19]. In comparison, the reported sensitivity for advanced pre-cancers by FIT in these series was 22% (14–33%) to 33% (23–43%) [18, 19]. When serrated lesions were considered separately, sensitivity for lesions ≥ 1 cm was 38% (16–64%) to 42% (33–53%) for MT-sDNA and 5% (2–12%) to 7% (1–26%) for FIT [17, 19]. MT-sDNA sensitivity is associated with size and advancing histology such that 63% (35–85%) of lesions ≥ 2 cm, 75% (19–99%) of lesions ≥ 3 cm, and 69% of lesions with high-grade dysplasia were detected [18••].

**Table 2:** Performance of MT-sDNA versus FIT

Study	Test	Specificity	Sensitivity by screen-relevant lesion type									
			All CRC	*P* value	Curable CRC*	*P* value	Proximal CRC	*P* value	Advanced pre-cancers	*P* value	Sessile serrated lesions ≥ 1 cm	*P* value
Imperiale et al. 2014 [17••]	MT-sDNA	87/90%^†^	92% (60/65)	0.002	93% (56/60)	0.002	90% (27/30)	0.04	42%** (321/757)	< 0.001	42.4% (42/99)	< 0.001
	FIT	95/96%^†^	74% (48/65)		73% (44/60)		67% (20/30)		24%** (180/757)		5.1% (5/99)	
Redwood et al. 2016 [18••]	MT-sDNA	91/93%^†^	100% (10/10)	0.48	n/a		n/a		41%^††^ (31/76)	0.006	38% (6/16)	0.07
	FIT	94/96%^†^	80% (8/10)		n/a		n/a		22%^††^ (17/76)		6% (1/16)	
Bosch et al. 2019 [19••]	MT-sDNA	89%	86% (6/7)	1.0	n/a		n/a		48%^††^ (44/92)	< 0.001	41% 11/27	0.02
	FIT	93%	100% (7/7)		n/a		n/a		33%^††^ (30/92)		7% 2/27	

*Stages 1–3

**Advanced adenomas and sessile serrated polyps ≥ 1 cm

^†^Specificity calculated from completely negative colonoscopies was higher than when patients with small polyps or other non-neoplastic findings were included among negative colonoscopies

^††^Advanced adenoma only

All 3 studies used a cross-sectional design; in each, a single FIT measurement was more specific (93% (91–95%) to 95% (94–95%)) than a single MT-sDNA test (87% (86–87%) to 91% (88–93%)) [17–19]. This difference initially raised concerns that MT-sDNA would lead to additional risks by increasing the rate of unnecessary colonoscopies. Whereas FIT requires annual measurement, MT-sDNA is measured every 3 years. Thus, the nominal false positive rate of 5% generated by each round of FIT screening is compounded over 3 years to 15% but MT-sDNA false positives remain at 13%. There is now some consensus that the specificity of FIT and MT-sDNA is likely to be similar over a 3-year period of programmatic test use [28••]. It is important to note that specificity was calculated for FDA labeling by defining a stool test–negative result from colonoscopies at which either non-advanced adenomas, hyperplastic polyps, non-neoplastic diseases, or no pathology was found; for both MT-sDNA and FIT, specificity is higher when calculated from colonoscopies which were negative for any of those findings (Table 2). It is further anticipated from a recent report that a second-generation MT-sDNA test will demonstrate even stronger specificity at 92% (88–94%) even when calculated from colonoscopies lacking only advanced neoplasia [29].

## Patients with False Positive Stool Screening Tests

New data have also emerged to guide clinicians on the management of patients with false positive stool tests. Currently, no diagnostic follow-up is recommended after false positive FIT on the basis of low diagnostic yield and high cost [30–32]. What about MT-sDNA? Does the DNA component of this stool test imply greater risk for subsequent aerodigestive tract (gastrointestinal, pulmonary, or head/neck) cancers in the future? In the past year, Berger and colleagues released a long-term follow-up study of patients with negative colonoscopy; over 200 patients had positive MT-sDNA results and over 1000 were negative. All were enrolled during the DeeP-C study and thus were all at average CRC risk. After a median of 5.3 years of follow-up, there were 5 aerodigestive cancers in the false positive group and 11 cancers in the true negative group (*P* = 0.15) [33••]. These findings corroborate recommendations by the Multi-society Task Force in their 2017 CRC screening guidelines to avoid further testing in patients with MT-sDNA followed by negative high-quality diagnostic colonoscopy [28••].

## Stool Testing Influences Diagnostic Colonoscopy Yield

Post-market studies demonstrate the high sensitivity of MT-sDNA for advanced pre-cancers, even among those who had undergone prior screening colonoscopy, and have also demonstrated particularly high yield in the detection of proximal lesions [34, 35]. This is of particular importance given that most interval colorectal neoplasia is right sided [36]. FIT sensitivity for advanced adenomas [37•] and cancers [17••] is biased toward the detection of left-sided lesions.

Both FIT and MT-sDNA testing also appear to influence the behavior of the physician at the time of diagnostic colonoscopy. Adenoma detection is significantly higher after a positive FIT in comparison to a similar group of patients undergoing screening colonoscopy alone [38•]. This trend has also been demonstrated after a positive MT-sDNA with a particular increase in detection of lesions in the proximal colon and substantially longer withdrawal times [39••]. Emerging data also suggest that MT-sDNA improves the detection of serrated polyps at diagnostic colonoscopy [40], an endpoint which was not shown after positive FIT [38•].

## Comparative Effectiveness by Computer Modeling

The United States Preventive Services Task Force (USPSTF) has endorsed several methods for CRC screening; hierarchy among screening strategies was not used in the 2016 guideline update [10••]. The guideline statements aimed to portray an overall balance of the relative benefits and harms to screening and were predominantly based on modeling studies. Modeling is particularly useful as there are few randomized trials with mortality or incidence endpoints; these endpoints have not been measured for all screening options, and it is impractical (if not impossible) to directly measure benefits and harms over a person’s lifetime.

For these estimates, the USPSTF commissioned a study using 3 validated microsimulation modeling platforms (Simulation Model of CRC, Microsimulation Screening Analysis for CRC, and CRC Simulated Population Model for Incidence and Natural History) [10, 41]. The primary measures of benefit were life-years gained and CRC deaths averted in comparison to no screening. Harms included (1) the anticipated number of gastrointestinal or cardiovascular complications attributable to colonoscopy, either for screening or in diagnostic follow-up of stool-based strategies, and (2) the total number of anticipated colonoscopies required for evaluation of positive stool-based strategies [41•]. Each model simulated the natural history of CRC based on the adenoma-carcinoma sequence and outcome is influenced by the respective sensitivity and specificity for the screening test used (derived from single test performance) [41•]. The greatest gain in life-years was with colonoscopy screening every 10 years; however, the gains over those achieved by non-invasive testing were surprisingly slim [41•]. Life-years gained through annual FIT were estimated to be 90% of those gained by colonoscopy and MT-sDNA life-years gained were roughly 93% of those obtained by annual FIT [10••]. Harms from either non-invasive strategy were substantially lower than those attributed to colonoscopy. The number of diagnostic colonoscopies required for the MT-sDNA strategy (1827) was *fewer* than that anticipated for the FIT strategy (1899), confirming the programmatic similarity in specificity from MT-sDNA, compared to FIT [10], as reviewed (above).

It is important to note that the models excluded the serrated polyp pathway that accounts for a third of CRC [42] and did not account for sensitivity variation based on polyp location [41•]. The models also used performance estimates for colonoscopy that assumed colonoscopy sensitivity based on tandem colonoscopy by expert operators that may be higher than suggested by recent studies showing high rates of variability among individual providers [43]. While imperfect, these methods have provided valuable insight into how these tests may perform. Plainly stated, MT-sDNA and FIT show highly comparable efficacy relative to colonoscopy under optimal colonoscopy conditions and with perfect test adherence.

## FIT and MT-sDNA Adherence

In the real world, adherence either to colonoscopy or non-invasive CRC screening is imperfect. Outside the USA, FIT is the dominant option used for population-wide CRC screening. The relatively high upfront price of MT-sDNA (approximately $500) and colonoscopy relative to FIT is a major adoption barrier. However, less than 50% of patients who have a FIT ordered will subsequently complete the test on the first round and adherence diminishes with each annual interval [44••]. Providers and systems that use FIT for CRC screening have improved adherence by providing patient support programs, at substantial cost; this is estimated to be over $150 per testing cycle in the USA [45]. How do these indirect costs and imperfect adherence rates impact cost-effectiveness? A Markov model of average-risk CRC screening was used to compare variable participation and assess cost-effectiveness of screening with MT-sDNA versus FIT or colonoscopy [45]. While all 3 options are cost-effective relative to no screening, colonoscopy and FIT are the dominant strategies, in this model, assuming perfect adherence. However, MT-sDNA becomes the dominant strategy if test adherence is 1.7-fold higher than FIT. Real-world manufacturer data show an overall 68% adherence with MT-sDNA, which may be as high as 71% in Medicare beneficiaries [46]. Up to 40% of patients using MT-sDNA testing have reported participation in screening for the first time and a substantial number of these appear to be overdue to initiate screening by 10 years or more [35••]. This favorable rate of intent-to-screen participation may be a result of a manufacturer-provided compliance program available to all patients for whom MT-sDNA has been ordered at no additional cost to patient or provider [46].

## Plasma-Based Colorectal Cancer Screening

While MT-sDNA testing appears highly acceptable to an increasing number of patients, screening for CRC with a blood sample may be a more attractive option for some. For this approach to be effective, it must show significantly greater adherence and exceed the current performance bar set by stool-based testing by either FIT or MT-sDNA for both CRC and precursors. As of early 2020, only one blood plasma test, methylated *SEPT9*, has been approved by the FDA. Prior to clinical availability of either test, prototypes of MT-sDNA and *SEPT9* tests were run in blinded fashion on matched stool and blood samples from 30 CRC cases, 22 advanced pre-cancers, 49 plasma controls, and 46 colonoscopy-negative stool controls. *SEPT9* detected 60% of CRCs, detected 14% of pre-cancers, and was positive in 27% controls. In contrast, MT-sDNA was positive in 87% CRCs, was positive in 82% of precursors, and had only a 7% false positive rate [47]. In the screening-setting study leading to FDA approval, methylated *SEPT9* test was performed on 7941 patients prior to blinded colonoscopy; there was no comparison to stool testing. Across all stages of CRC, *SEPT9* was 48% sensitive and almost 92% specific but detected only 11% of advanced pre-cancers [20]. When reviewed by USPSTF, the Task Force expressed concern that *SEPT9* was not likely to be comparably effective to other options due to low sensitivity for CRCs and precursors [20]. In a more recent study that mixed referred cases and a prospective screening arm, a re-configured *SEPT9* test was non-inferior to FIT for CRC sensitivity (72% and 68%, respectively) but at substantially lower specificity (97% and 81%, respectively) [48].

Several alternative blood-based assays are in development to advance non-invasive CRC screening via a “liquid biopsy.” For example, Colvera® by ClinicalGenomics (Bridgewater, NJ) is a plasma test of methylated DNA that is designed for surveillance after treatment of CRC; it is being evaluated for CRC screening but the adenoma sensitivity is 9% [49•]. Cell-free DNA coupled with machine learning is also being investigated but is currently demonstrating detection only after the development CRC [50•]. CancerSEEK® by Thrive Earlier Detection Corp. (Cambridge, MA) aims to increase early cancer detection through utilization of protein biomarkers in addition to tumor genetic alterations [51•]. CancerSEEK® is also being designed to detect several different cancer types via a single blood sample. When evaluated among 1005 patients diagnosed with stage I to III ovary, liver, stomach, pancreas, esophagus, colorectal, lung, or breast cancer, the median sensitivity for stage I cancers combined was 43% [51•]. Replication of these results has not yet been reported. More recently, an analysis from the Circulating Cell-free Genome Atlas (CCGA) study (NCT02889978) sponsored by GRAIL (Menlo Park, CA) also reported on sensitivity for plasma-based detection of early cancers, including CRC, where sensitivity was greater than 50% for stage I and greater than 75% for stages II–III [52].

**Fig. 1:**
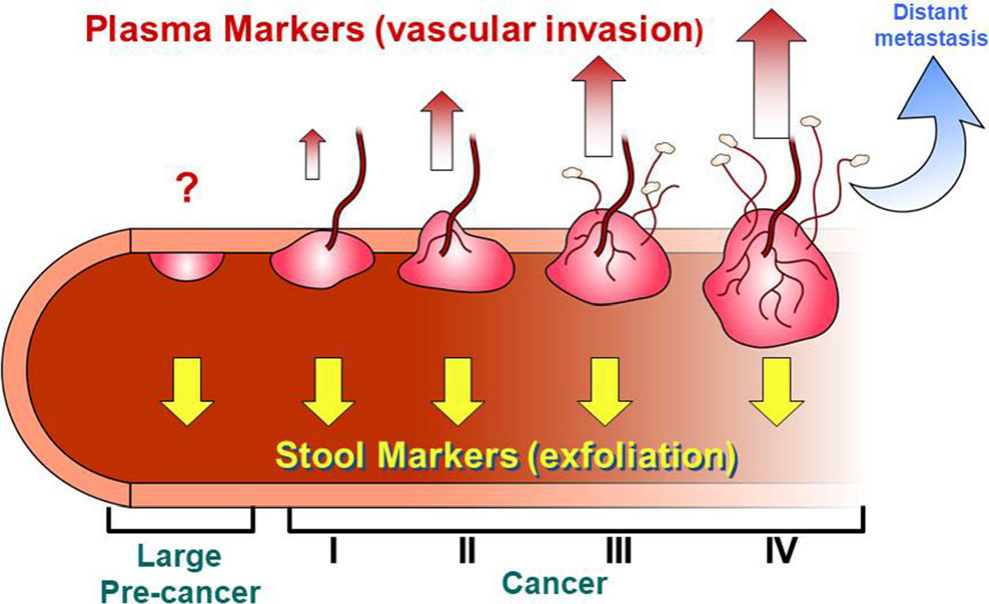
Conceptual model in progression of colorectal tumor marker generation and subsequent detection through stool exfoliation versus vascular invasion and subsequent plasma detection. Adapted with permission. Ahlquist DA, Taylor WR, Mahoney DW, Zou H, Domanico M, Thibodeau SN, et al. The stool DNA test is more accurate than the plasma septin 9 test in detecting colorectal neoplasia. *Clin Gastroenterol Hepatol*. 2012;10(3):272-7.e1

Despite these promising results, it is critical to highlight that there is no blood sample test available at this time with prospectively validated sensitivity for advanced adenomas equivalent to either FIT or MT-sDNA in the screening setting. We hypothesize that plasma-based detection of pre-cancers is likely to remain sub-optimal on the basis of biological factors rather than assay sensitivity or marker optimization. Precursors to CRC develop among epithelial cells confined to the colonic mucosa. By definition, these lesions lack a direct blood supply, as vessels are submucosal structures and not in direct cellular approximation to colorectal neoplasia until invasive cancer occurs (Fig. 1) [53]. In contrast, exfoliation of neoplastic cells and cellular debris into the luminal mucocellular layer has been shown to be continuous and abundant from both CRC and precursor lesions [26]. Without detection of advanced precursors equivalent to or superior to stool-based tests, plasma tests are not anticipated to be effective in reducing CRC incidence.

## Conclusion and Future Directions

Widespread screening for CRC has reduced incidence and mortality of this devastating disease but remains short of public health goals [11]. The relatively recent expansion of non-invasive strategies for screening will not eliminate the need for colonoscopy but may increase population participation in screening [14]. Encouragingly, several studies have also suggested a potential for added benefit by the utilization of non-invasive tests for screening in that diagnostic colonoscopy may be performed with higher quality when physicians are aware of the positive stool test result [38•, 39••]. Observed increases in adenoma detection at colonoscopy [54] after stool testing and advanced adenomas detected by MT-sDNA after negative colonoscopy [40] are both anticipated to reduce the rate of post-colonoscopy interval CRC [43], particularly in the proximal colon. These emerging concepts, and the use of interval CRC as a clinically impactful endpoint, appear highly deserving of prospective evaluation.

## Abbreviations

CRC: Colorectal cancer
CI: Confidence interval
FIT: Fecal immunochemical test
gFOBT: Guaiac-based fecal occult blood testing
MT-sDNA: Multi-target stool DNA
*SEPT9*: Methylated *SEPTIN9*
USPSTF: United States Preventive Services Task Force
